# The Antimicrobial Effect of Silver Ion Impregnation into Endodontic Sealer against *Streptococcus mutans*

**DOI:** 10.2174/1874210600802010018

**Published:** 2008-02-21

**Authors:** J. Kreth, D. Kim, M. Nguyen, G. Hsiao, R. Mito, M.K. Kang, N. Chugal, W. Shi

**Affiliations:** 1 UMN School of Dentistry, Minneapolis, MN 55455; 2 UCLA School of Dentistry, Los Angeles, CA 90095; 3 UCLA Jonsson Comprehensive Cancer Center, Los Angeles, CA 90095; 4 David Geffen School of Medicine at UCLA, Los Angeles, CA 90095, USA

**Keywords:** ontic sealer, silver, root canal, *Streptococcus mutans*

## Abstract

Pulpal and periradicular diseases are primarily caused by bacterial invasion of the root canal system as a result of caries progression. The presence of residual bacteria at the time of root canal completion (obturation) is associated with significantly higher rate of treatment failure. Re-infection of obturated root canals can be potentially prevented by enhancing the antibacterial activities of root canal obturation materials. We evaluated, in an *in vitro* model, the antimicrobial efficacy of silver ions added to a common endodontic sealer. For that purpose we performed growth inhibition studies and bacterial viability tests. We measured the zone of inhibition, optical density and performed confocal laser scanning microscopy. Our results show that the silver ions enhance the antimicrobial activity of the root canal sealer against *Streptococcus mutans*. This study approach may hold promise for studying other biologically based therapies and therefore increasing the success rate of routine orthograde root canal treatment.

## INTRODUCTION 

Numerous studies have shown that bacteria are the main etiologic agent of pulpal infection and periradicular lesion formation [1-4]. Most frequent route of infection is the progression of the carious lesion into the pulp. Other routes of pulpal infections include cracks, trauma, periodontal pockets and anachoresis. The primary bacterium associated with dental caries is *Streptococcus mutans* [5]*. *However, bacteria associated with deep caries and advanced root carious lesions belong to the group of Gram-positive cocci and rods, and are dominated by streptococci, lactobacilli and *Actinomyces* [6-8]. On the other hand, the microbial flora of infected root canals is polymicrobial in nature and is dominated by Gram-negative anaerobes [9, 10]. Therefore, microbial selection occurs within the root canal ecosystem associated with infections of endodontic origin. Importantly, we can not rule out that this microbial selection followed distinctive steps initiated by *S. mutans*, which might create an environment, including initial lesions of the enamel, favorable for the colonization of the aforementioned bacterial groups.

Studies on endodontic prognosis have demonstrated that the presence of residual bacteria at the time of root canal completion (obturation) is associated with significantly higher rates of treatment failure [11]. In addition, endodontically treated tooth is vulnerable to recontamination from the oral cavity. A recent study [12] demonstrated the presence of biofilms in otherwise well executed root canal treatment. Therefore, the endodontically treated tooth is vulnerable to treatment failure due to the residual infection as well as the re-infection of the root canal system.

The core concept of endodontic treatment is therefore directed at thorough chemo-mechanical debridement and biomechanical preparation, followed by a quality obturation of the root canal system. Ideally, the antibacterial phase of root canal treatment should be completed by the time of root canal obturation [13]. Successful healing of periradicular disease therefore depends not only on removal of the bacteria from the root canal system through chemo-mechanical debridement, but also on preventing re-infection through a good root canal obturation [14-16]

With the currently available root filling materials, even an ideally obturated root canal is susceptible to re-infection by microorganisms introduced into the system *via *coronal leakage [14-17]. The commonly used root filling material, gutta percha, does not adhere to dentin in the absence of root canal sealer [18]. Therefore, the sealer is necessary to prevent leakage along the gutta percha and dentin interface to prevent ingress of the bacteria from the oral environment to the periapical tissues [19]. In addition, the sealer is also more likely to come in direct contact with the remaining viable microorganisms in the dentinal tubules and undebrided parts of the root canal system [12].

To improve the prognosis of endodontically treated teeth, we considered incorporating antimicrobial agent(s) into the endodontic sealer. Such sealer ought to be antimicrobial, non-toxic, non-immunogenic and biocompatible to the human patient.

It is known that certain metal ions have a negative influence on bacterial enzymatic functions, e.g. silver ions inhibits fructosidase from *Streptococcus mutans* [20], or on cellular processes like the adaptive response caused by DNA damaging processes as demonstrated for *Escherichia coli* and cadmium/mercury ions[21]. Although the efficacy of silver ions as anti-infection and anti-contamination agent is well established [22], the mechanism by which the silver ions exerts its antibacterial activity is not yet fully understood. Silver ions have been shown to have broad spectrum antibacterial and antifungal activity [23, 24], and also work well under anaerobic conditions [25]. Silver ions have also been shown to destabilize biofilm structures allowing for increased susceptibility of the bacteria to antimicrobial agents [26]. Recently, silver ions have been placed in dental restorative materials for its slow releasing antimicrobial activity [27].

Silver ions nonspecific antimicrobial activity may be utilized to improve the microbiocidal efficacy of endodontic sealers against the remaining bacteria in the root canal system that survive rigorous chemo-mechanical treatment. Bacterial re-infection of the root canal system after endodontic treatment may also be retarded by the continual release of silver ions from the root canal sealer. To study this potential application of silver ions, in a first series of experiments, we tested the antimicrobial activity of silver ions against *S. mutans*

In this pilot study, we tested the antimicrobial activity of silver ions incorporated into a commonly used endodontic sealer. We chose *S. mutans* because its cariogenicity has been well established [5, 28] and because the effect of silver ions on the metabolism of *S. mutans* has been demonstrated [20]. Although *S. mutans* is isolated occasionally from infected root canals with apical periodontitis [29, 30], it is an appropriate bacterium to use in this study model because it is amenable for growth in the laboratory settings, unlike some of the strict anaerobes isolated from root canals and susceptible to genetic manipulations. Additionally, the virulence properties of *S. mutans* are well described and the biology of this organism is well established, which allows us to further define the antimicrobial activity of silver ions.

Therefore, the purpose of this study is to test the antimicrobial efficacy of silver ions against the oral bacterium *S. mutans*, when incorporated into the root canal sealer.

## MATERIAL AND METHODS

### Bacterial Strains, Media and Chemicals

S. mutans strain UA159 [31] was maintained on brain heart infusion (BHI, Difco Laboratories, Sparks, MD] agar plates or liquid media. The bacteria were incubated in an anaerobic chamber (90% N2; 5% CO2; 5% H2) at 37ºC. Silver sodium hydrogen zirconium phosphate (silver powder) was obtained from Sterisil (Palmer Lake, CO). Silver containing endodontic sealer plus eugenol (Kerr sealer) used was Kerr Pulp Canal SealerTM EWT (Kerr USA, Romulus, MI).

### Study Design and Variables

We evaluated the antimicrobial efficacy of the silver ions added to the endodontic silver containing sealer. The performed experiments were aimed to be consistent in the growth medium used. Variables included diffusion limitation through agar *vs.* liquid cultures, as well as time frames used for the inspections of the effect of silver-ion on the growth and viability of *S. mutans. *The following experiments were designed:

#### 1. Evaluation of the Antimicrobial Efficacy of Silver Ions in Suspension Against S. mutans

Brain heart infusion (BHI) agar plates in 100-mm Petri dishes were divided into 4 quadrants, and a 3 mm diameter well was cut out in each quadrant. Silver ion suspensions in concentrations of 1 mg/ml, 5 mg/ml, and 10 mg/ml were prepared by dissolving appropriate amounts of silver sodium hydrogen zirconium phosphate in sterile water. Sterile water without silver powder served as control. A 30 μl aliquot of each solution was placed into the agar wells, and a BHI soft agar overlay culture of *S. mutans* containing 1.0 x 10^5 ^CFU/ml was poured on top of the BHI agar plates. Plates were allowed to set and subsequently incubated at 37(C in an anaerobic chamber for 24 hours. The diameter of the zone of inhibition was measured in millimeters (mm).

#### 2. Evaluation of the Antimicrobial Efficacy of Silver Ions Incorporated into the Kerr Pulp Canal SEALER by Agar Diffusion Assay

Control discs were prepared by addition of 65 mg Eugenol to 250 mg Kerr sealer powder. Experimental discs were prepared in the following manner: Silver sealer discs (5%) contained 237.5 mg Kerr powder, 12.5 mg silver powder, and 65 mg eugenol; Silver sealer discs (10%) contained 225 mg Kerr powder, 25 mg silver powder, and 65 mg eugenol; Silver sealer discs (20%) contained 200 mg Kerr powder, 50 mg silver powder, and 65 mg of eugenol. Ten millimeters (10 mm) diameter well were cut out of a 1 mm thick Modern Pink No. 3 Wax, Type I, sheet (Heraeus Kulzer Inc, Armonk, NY), which served as molds for preparation of silver discs. Once the Kerr sealer preparations were mixed together, the mixture was carefully placed into the wells, and was allowed to set at 37(C for 24 hours. The discs were then placed on BHI plates and overlaid with bacterial soft agar containing *S. mutans* using the same method described above. Plates were subsequently incubated at 37(C in an anaerobic chamber for 24 hours. The diameter of the zone of inhibition was measured in millimeters (mm).

#### 3. Evaluation of the Antimicrobial Efficacy of the Silver Ions Incorporated into the Kerr Sealer against Planktonic Bacteria

A control sealer was prepared with 800 mg Kerr sealer powder and 200 mg eugenol. The experimental Kerr sealer containing silver powder (20%) was prepared by mixing 750 mg Kerr powder, 50 mg silver powder, and 200 mg eugenol. The mixtures were poured into 30-mm Petri dishes. The Petri dishes were kept at 37(C for 24 hours to allow for complete setting of the sealer. After the sealer was set, the Petri dishes were sterilized with UV light for 1 hour. A 500 ml beaker containing 50 ml BHI broth was inoculated with a 1:30 dilution of an overnight *S. mutans* culture. The sterilized Petri dish was placed in the 500 ml beaker. The beaker containing *S. mutans* liquid culture and the Petri dish with the sealer preparation was sealed with a sterile piece of aluminum foil. This apparatus was placed in the anaerobic chamber and after overnight incubation the terminal absorption was measured at 600 nm spectrophotometrically. 

#### 4. Microscopic Evaluation of Bacterial Viability after Short Term Contact with the Kerr Sealer Disks Containing Added Silver Ions

The Kerr sealer discs were prepared with or without 20% silver ion according to the methods described for the agar diffusion assay. A 100 μl of an overnight *S. mutans* culture was placed on top of the sealer discs and incubated in the anaerobic chamber for 85 min and 190 min. After incubation, an aliquot of the bacterial solution (5 μl) was drawn from the top of each sealer disc and placed on a microscope slide. Confocal laser scanning microscopy (CLSM) was performed using LSM 5 PASCAL with LSM 5 PASCAL software (Carl Zeiss, Jena, Germany). The microscope was equipped with the detectors and filter sets for monitoring of the green and red fluorescence. Images were obtained with a 40x1.3 Plan-Neofluar oil objective. The bacterial cells were stained with the LIVE/DEAD^®^ BacLight Bacterial Viability Kit (Invitrogen, Carlsbad, CA) following the manufactures instructions.

### Statistical Methods

Statistical analysis of two data sets was performed using QuickCalcs online calculators (http://www.graphpad.com/quickcalcs/index.cfm) using the *t*-test software presenting mean and S.D. Statistically significant differences were set at a *P*-value of < 0.05. Differences between group means (silver ion concentrations) were tested with ANOVA followed by Tukey's test.

## RESULTS

### Antimicrobial Efficacy of Silver Ions in Suspension against *S. mutans*

**Fig. (1). Antimicrobial effect of silver ions.**
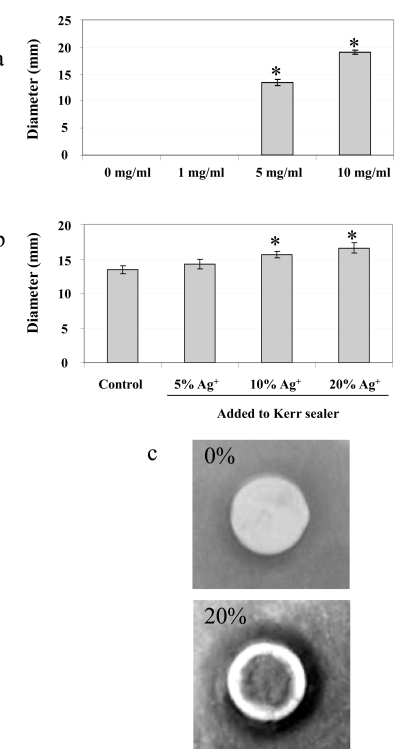
a) Inhibition of *S. mutans* growth in the presence of silver ions dissolved in suspension. b) Growth inhibitory effect of silver ions in agar diffusion assay at various concentrations. c) Close-up photograph of an overlay assay demonstrating the inhibitory effect of Kerr sealer impregnated with 20% silver ions compared to Kerr sealer alone.^ *^ Denotes a significant difference in the growth inhibition (P < 0.05), see text for details. n = 4.

The antimicrobial effect of silver ions in suspension was tested on *S. mutans* in an overlay assay performed on BHI plates. Visualization of the zone of inhibition was used to determine the antimicrobial efficacy of silver ions at various concentrations. The diameter (mm) of the inhibitory zone was measured after 24 hrs incubation (Fig. **1a**). The solution containing 0 mg/ml or 1 mg/ml of silver ions showed no inhibition. A statistically significant difference in the zone of inhibition was observed for 5 mg/ml and 10 mg/ml (*P* < 0.05, by ANOVA), respectively.

### Antimicrobial Efficacy of Silver Ions Incorporated into the Kerr Sealer Measured by Agar Diffusion Assays against *S. mutans*

The results of these experiments demonstrated that the Kerr sealer discs without additional silver ions (control) had the smallest inhibition zone (Fig. **1b**). Increasing diameters of inhibition were observed with the addition of higher silver ion concentrations. Kerr discs containing an added 20% concentration of silver ions showed the greatest zone of inhibition followed by a 10% concentration. Both concentrations showed a statistically significant difference when compared to that of 5% silver ion concentration (*P*< 0.05, *t*-test). These results indicate that the antimicrobial effect of silver ions is conserved, compared to the liquid suspension assay described above, when it is incorporated into the Kerr sealer.

### Antimicrobial Efficacy of the Silver Ions Incorporated into the Kerr Sealer against Planktonic Bacteria

**Fig. (2).**
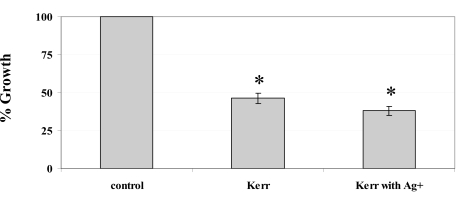
Percentage growth inhibition in liquid culture. The terminal optical density after growth in 50 ml BHI medium was determined with respect to that of the unexposed control cells (set to 100%). ^*^ Denotes a significant difference in the growth inhibition (P < 0.05 by ANOVA). n = 3.

We also tested the antimicrobial effects of silver ions incorporated into the Kerr sealer against planktonic cultures of* S. mutans*. The terminal optical density (TOD) of the *S. mutans* planktonic cultures was measured after 24 hours incubation to determine the percentage of inhibition compared to the control cells not exposed to any sealer. A statistically significant difference of the TOD was measured when the cells were exposed to the Kerr sealer containing 20% silver ions, followed successively by the cells exposed to Kerr sealer alone and the control cells (Fig. **2**), (*P* <0.05, by ANOVA).

### Bacterial Viability after Short Term Contact with the Kerr Sealer Disks Containing Added Silver Ions

**Fig. (3).**
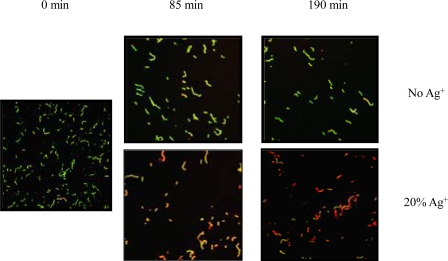
Bacterial viability determined with the *Bac*Light staining kit after incubation of *S. mutans* on Kerr sealer discs with or without 20% silver ion. Green stain represents viable bacteria. Red stain represents membrane-compromised bacteria. Magnification 400x. The micrograph is a representative of two independent tests done on different days

The bacteriocidal effect of the silver ions impregnated Kerr sealer was observed with CLSM after a short term exposure to the silver ions impregnated disks. For this purpose, *S. mutans* cells were incubated on the surface of the Kerr sealer discs containing 20% silver ions and the cell viability was determined using the LIVE/DEAD^®^ *Bac*Light bacterial viability kit. The LIVE/DEAD^®^ *Bac*Light bacterial viability kit is widely used for the enumeration of bacteria and provides information about the viability of bacterial cells [32] . The kit contains the fluorescent stains SYTO 9, which penetrates most membranes freely, and propidium iodide, which is highly charged and normally does not permeate cells but does penetrate the cells with compromised membranes.Simultaneous staining with both dyes therefore results in green fluorescence of viable cells with intact membranes, whereas the cells with compromised membrane integrity show intense red fluorescence. After 85 and 190 min incubation, the cell suspensions were removed and several fields were examined to look for consistency. Cells cultured on the Kerr sealer disc containing 20% silver ions showed increased loss of membrane integrity compared with those on the Kerr sealer alone (Fig. **3**). These results demonstrate effective bactericidal effects of Kerr sealer with silver ion impregnation after a short term exposure to *S. mutans*.

## DISCUSSION

Eradication of microorganisms from an infected root canal system has been demonstrated, in many studies, to be the key to successful endodontic treatment and favorable prognosis. Therefore, endodontic treatment is focused on the asepsis and disinfection of the root canal system as well as the preservation of the remaining tooth structure [33]. However, microorganisms can still survive rigorous endodontic disinfection protocols [12]. Extensive research in the area of endodontic materials demonstrates that complete sealing of the root canal system with currently accepted materials and obturation techniques is not a predictable procedure [17]. Numerous studies have demonstrated extensive coronal leakage and bacterial recontamination of the root canal space along the root canal fillings in experimental models *in vitro* [15, 34, 35]and *in vivo* [16] . Microorganisms that infect root canals may adhere to the dentinal wall or penetrate deeper into dentinal tubules [7], whereas the bacteria that adhere superficially to the root canal walls are more likely to be eradicated by chemo-mechanical debridement. However, bacteria that infect dentinal tubules [7] and remain in undebrided parts of the root canal system [12] may be susceptible to the antimicrobial components leaching from the sealer. Therefore, root canal sealers with excellent adhesion to dentin and sustained antibacterial activity are desired to entomb and/or kill the microorganisms that survive chemo-mechanical debridement.

This study demonstrates the antimicrobial effects of silver ions against the oral bacterium *S. mutans* under several experimental conditions. Although *S. mutans* is not considered the primary endodontic pathogen, it was an appropriate bacterium to use in this study model because the virulence properties of *S. mutans* are well described and the biology of this organism is well established. This allows us to address, for example, the antimicrobial activity of silver ions against this microorganism under the experimental setup described in this study in the future experiments. Furthermore, this study is in good agreement with the previous findings which showed effective antimicrobial activity of liquid-solubilized silver ions [36] against various oral pathogens. Our results also demonstrated the ability of silver ions to dissociate from the solidified endodontic sealer and to exert its antimicrobial activity.

The addition of silver ions enhances the antimicrobial activity of the Kerr root canal sealer in the suspension culture during overnight incubation and in the short term contact over 190 min with *S. mutans*. Silver ions in liquid suspension showed marked antimicrobial effects at concentrations of 5 and 10 mg/ml while no effects were found at 1 mg/ml (Fig. **1a**), indicating the existence of a threshold level of silver ions to elicit the antimicrobial effects. The Kerr sealer contains silver in its original formulation, but this concentration (25 to 30% in the powder base, according to manufacturers MSDS) has no visible effect on *S. mutans* in the agar diffusion test. This is in agreement with the results published earlier, showing only discrete inhibitory effects of this sealer against a variety of Gram-positive and Gram-negative bacteria [37]. This could partly explain the marginal antimicrobial effect of the silver-impregnated Kerr sealer discs in the broth culture (Fig. **2**), since the silver ions released from the discs are significantly diluted in the broth media. The broth culture experimental model does not accurately represent the mode of antimicrobial activity of the silver ions impregnated into the root canal sealer. The sealer and leachable silver ions are in close contact with the endodontic pathogens in the root canal system, therefore the silver ions elicit their antimicrobial activity over a much shorter distance. However, the statistical analysis revealed a significant difference in the inhibition, demonstrating that the addition of silver ions to the Kerr sealer improves the antimicrobial activity against *S. mutans*.

The antimicrobial effects of the silver-impregnated Kerr sealer discs was markedly higher than that of the control sealer discs when the bacterial cells were in close contact with the sealer discs (Fig. **3**). There is a clear indication that the silver ions incorporated into the endodontic sealer effect the membrane integrity of *S. mutans*, which could lead to diminished viability of the bacteria.

Clinical implications of this *in vitro* study are noteworthy. Since the prognosis of endodontic treatment is closely related to the presence of microorganisms in the root canal system, the failure of treatment is more likely to occur if the bacteria are either incompletely removed or bacteria re-infect the root canal system. Potentially, the proximity of silver ions in the sealer to the remaining or invading bacteria may contribute to the increased success of the treatment.

We acknowledge that this study is not comprehensive in the scope and therefore cannot serve as the basis to recommend clinical application. Further studies are needed to address the efficacy of silver ions as well as silver ion impregnated sealer against common endodontic pathogens. In addition, testing for cytotoxicity, mutagenicity and other potential long-term effects of silver ions are recommended.

At the same time we recognize the potential of this study model for further studies of not only silver ions, but to explore possible application of bioactive molecules like antimicrobial peptides used recently in the killing of oral pathogens [38, 39] and to address the susceptibility of bacterial biofilms to these improved endodontic sealers.
